# A network-based approach reveals long non-coding RNAs associated with disease activity in lupus nephritis: key pathways for flare and potential biomarkers to be used as liquid biopsies

**DOI:** 10.3389/fimmu.2023.1203848

**Published:** 2023-07-05

**Authors:** George Sentis, Catherine Loukogiannaki, Nikos Malissovas, Dionysis Nikolopoulos, Theodora Manolakou, Sofia Flouda, Maria Grigoriou, Aggelos Banos, Dimitrios T. Boumpas, Anastasia Filia

**Affiliations:** ^1^Laboratory of Autoimmunity and Inflammation, Center of Clinical, Experimental Surgery and Translational Research, Biomedical Research Foundation Academy of Athens, Athens, Greece; ^2^4th Department of Internal Medicine, Attikon University Hospital, National and Kapodistrian University of Athens Medical School, Athens, Greece; ^3^1st Department of Internal Medicine, University Hospital of Alexandroupolis, Democritus University of Thrace, Alexandroupolis, Greece

**Keywords:** lupus nephritis, long non-coding RNAs, WGCNA, disease activity, ceRNA, blood-based biomarker, RNA-sequencing, SLEDAI-2K

## Abstract

**Objective:**

A blood-based biomarker is needed to assess lupus nephritis (LN) disease activity, minimizing the need for invasive kidney biopsies. Long non-coding RNAs (lncRNAs) are known to regulate gene expression, appear to be stable in human plasma, and can serve as non-invasive biomarkers.

**Methods:**

Transcriptomic data of whole blood samples from 74 LN patients and 20 healthy subjects (HC) were analyzed to identify differentially expressed (DE) lncRNAs associated with quiescent disease and flares. Weighted gene co-expression network analysis (WGCNA) was performed to uncover lncRNAs with a central role (hub lncRNAs) in regulating key biological processes that drive LN disease activity. The association of hub lncRNAs with disease activity was validated using RT-qPCR on an independent cohort of 15 LN patients and 9 HC. cis- and trans-targets of validated lncRNAs were explored *in silico* to examine potential mechanisms of their action.

**Results:**

There were 444 DE lncRNAs associated with quiescent disease and 6 DE lncRNAs associated with flares (FDR <0.05). WGCNA highlighted IFN signaling and B-cell activity/adaptive immunity as the most significant processes contributing to nephritis activity. Four disease-activity-associated lncRNAs, namely, NRIR, KLHDC7B-DT, MIR600HG, and FAM30A, were detected as hub genes and validated in an independent cohort. NRIR and KLHDC7B-DT emerged as potential key regulators of IFN-mediated processes. Network analysis suggests that FAM30A and MIR600HG are likely to play a central role in the regulation of B-cells in LN through cis-regulation effects and a competing endogenous RNA mechanism affecting immunoglobulin gene expression and the IFN-λ pathway.

**Conclusions:**

The expression of lncRNAs NRIR, KLHDC7B-DT, FAM30A, and MIR600HG were associated with disease activity and could be further explored as blood-based biomarkers and potential liquid biopsy on LN.

## Introduction

Systemic lupus erythematosus (SLE) is a chronic autoimmune disorder with manifestations of variable severity in multiple organs, predominantly affecting women of reproductive age (1). Lupus nephritis (LN), the renal manifestation of SLE, affects a significant proportion of patients and is accompanied by permanent organ damage and increased morbidity and mortality rates (2, 3). Initial diagnosis and monitoring of LN rely on invasive kidney biopsy (4). Current therapies are unable to suppress flares in more than half of LN patients, and residual inflammation is detected in cases of repeat biopsies of clinically inactive patients (5) with long-term disease leading to damage accrual (4). Considering the above and the fact that low disease activity is related to more favorable outcomes for SLE patients (6), it is essential to identify a non-invasive blood-based biomarker to reliably quantify disease activity in LN patients without the need for kidney biopsy.

Long non-coding RNAs (lncRNAs) are transcripts longer than 200 nucleotides either without coding potential or featuring small open reading frames (ORFs) that translate in peptides of insignificant length (7, 8). They have been known to partake in transcriptional regulation, affecting the expression of genes in their vicinity (*cis*-) or distant targets (*trans*-acting lncRNAs) (9), exerting their effect at a transcriptional, posttranscriptional, or chromatin modification level with repressive or inducing aftermaths (10). LncRNAs have emerged in the last years as promising biomarker molecules of prognostic or diagnostic value, mainly in the field of cancer research (11–13). Although their role is not yet clear in SLE, studies in autoimmunity have shown lncRNAs to be stable in human plasma samples; thus, they are suitable candidates for non-invasive biomarkers (14).

Previous studies have reported that lncRNAs are differentially expressed in SLE patients compared with healthy controls. In particular, by sequencing total RNA isolated from peripheral blood mononuclear cells (PBMCs), researchers identified over 1000 DE lncRNAs, and using qPCR experiments, they validated the results of seven transcripts located near lupus susceptibility loci (15). A second study profiled the expression of lncRNAs in kidney tissue samples of a murine model with LN identifying and validating DE lncRNAs (i.e., Neat1, Lincpint) and DE mRNAs (Tgfbr1, Riok3). The same study evaluated the co-expression of lncRNAs with neighboring mRNAs and validated five gene co-expression pairs, namely, Gm26601-Dip2c, 2500002B13Rik-Hmgb2, Gm26556-Ppp1r9a, 1700020N18Rik-Hes6, and Gm20513-’H2-Aa’ (16). A third study identified three lncRNAs (GAS5, linc0949, and lnc-DC) with stable plasma levels that are differentially expressed between healthy controls and LN patients. Interestingly, when comparing lnc-DC expression in non-nephritis SLE patients to healthy controls, they found the lncRNA to be upregulated, with contrasting results emerging in the LN patients vs. healthy controls comparison where the gene was found to be downregulated (17). This observation hints the existence of a biomarker, possibly based on the expression of an lncRNA, able to distinguish LN patients from non-nephritis SLE patients. Another group investigated the transcriptional landscape of non-coding RNAs in SLE showing the repressive capabilities of lncRNA ENSG00000236525 on the gene of C–C chemokine receptor type 7 (CCR7), ultimately affecting the differentiation of follicular helper T cells with an impact on autoimmunity (18). Finally, a study focusing specifically on lncRNA NEAT1 and its association with SLE revealed an upregulation of its expression in peripheral blood monocytes and an active role in the inflammation signaling of TLR4 (19).

Our study focuses on the association of lncRNAs with disease activity in LN. In this report, we describe the involvement of lncRNAs in flares of LN and propose lncRNAs with the potential to be used as liquid biopsy. We also suggest potential mechanisms for their involvement in this significant organ-specific manifestation of SLE.

## Materials and methods

### Sample collection

**Figure 1:**
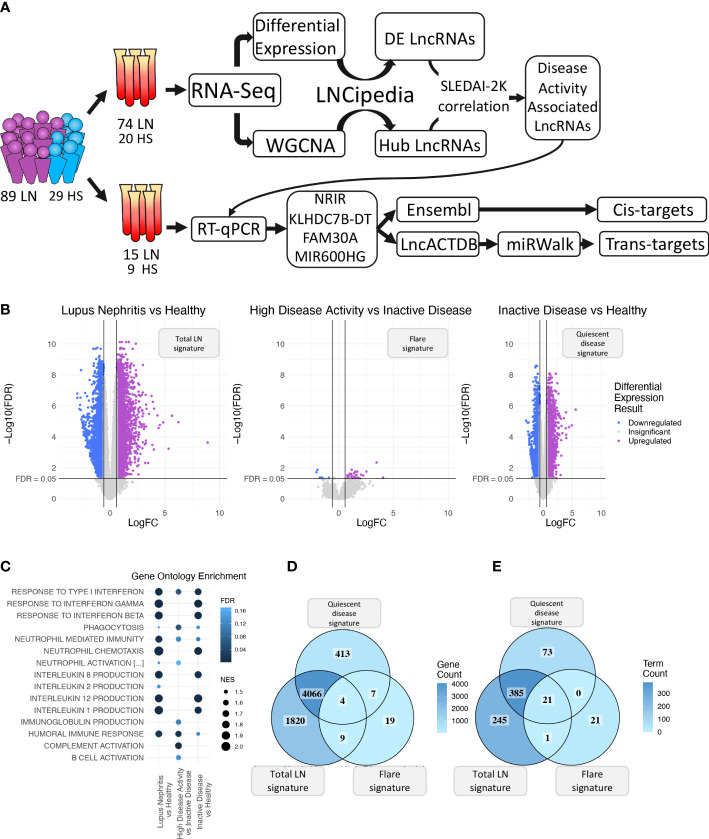
**(A)** Graphical overview of our research steps. **(B)** Volcano plot of the three differential expression analyses (DEA) performed comparing (left to right) Lupus Nephritis patients vs. Healthy controls, High Disease Activity vs. Inactive Disease patients, and Inactive Disease patients vs. Healthy controls. Upregulated genes are colored violet, and downregulated genes are colored blue. Genes not reaching our significance thresholds (|log2FC| >0.58 and FDR <0.05) are shown in gray. **(C)** Bubble plot showing inflammation-related Gene Ontology terms found as significantly enriched in each of the three DEA when performing gene set enrichment analysis (GSEA). Color represents FDR values whereas bubble size represents the Normalized Enrichment Score of each term. **(D)** Venn diagram comparing the DE genes of each DEA. Color gradient corresponds to the gene count in each compartment. **(E)** Venn diagram comparing the enriched terms of each GSEA. Color gradient corresponds to the term count in each compartment.

Samples from 74 LN patients and 20 healthy subjects (HC) served as our Discovery Cohort (**Figure 1**, **Supplementary Table 1**) (20). Patient samples either had active LN at the time of sampling (n = 34) or had a previous manifestation of LN over the course of the disease (n = 40). Active LN was defined as previously described (5). An independent cohort of 15 LN patients and 9 HC were used as the Validation Cohort (**Supplementary Table 2**). LN samples were split in three groups according to their clinical SLE disease activity index (SLEDAI-2K) (21). The groups were defined as Inactive Disease (InaD) (SLEDAI-2K: 0-4), Intermediate Disease Activity (IDA) (SLEDAI-2K:5-11), and High Disease Activity (HDA) (SLEDAI-2K:12+) groups.

### Library construction

Whole-blood extracts of the Discovery Cohort were total RNA-sequenced. PAXgene Blood RNA Kit IVD (#762174, Qiagen) and Tempus™ Spin RNA Isolation Kit (#4380204, Thermo Fisher Scientific) were used for RNA isolation. Library construction was performed using NEBNext^®^ rRNA Depletion Kit v2 (#E7400, New England Biolabs) and NEBNext^®^ Ultra^™^ II Directional RNA Library Prep with Sample Purification Beads Kit (#E7765 New England Biolabs). Library quality was assessed using a 2100 Bioanalyzer (Agilent), and a Qubit 4 Fluorometer with dsDNA HS assay kit (#Q32854, Thermo Fisher Scientific) was used for quantitation of libraries. 100-bp paired-end sequencing was performed on an Illumina Nova-Seq 6000 System.

### Sequencing QC and analysis

Quality of sequencing data was assessed using FastQC software (version:0.11.9, RRID : SCR_014583) (22). Adapter sequences and low-quality bases (Q<30) of the 3′ end were trimmed using Cutadapt (v:1.18, RRID : SCR_011841) (23), and trimmed reads were aligned to the human reference genome (v:hg38) with GENCODE annotation (v:39, RRID : SCR_014966) (24) using STAR (v:2.6.1b, RRID : SCR_004463) (25). Samtools (v:1.9, RRID : SCR_002105) (26) was used to sort bam files, and HTSeq (v:0.11.0, RRID : SCR_005514) (27) was used to extract gene expression counts.

### Differential expression analysis

Raw counts were normalized and analyzed using edgeR (28) package (v:3.38.1, RRID : SCR_012802) in R (29) (v:4.2.0, RRID : SCR_001905) to identify mRNAs and lncRNAs that are differentially expressed (DE) between (a) all LN patients and healthy controls (HC) (DEmRNAs, DElncRNAs), (b) HDA and InaD groups, and (c) InaD and HC. Genes were considered DE when |FC| >1.5 and FDR <0.05. Results were visualized using ggplot2 (v3.4.1, RRID : SCR_014601) (30).

### Gene set enrichment analysis

Preranked gene-set enrichment analysis (GSEA) against Gene Ontology Biological Process (GO : BP MSigDB (31) v2022.1.Hs, RRID : SCR_016863) terms was performed using GSEA (32) software (v:4.2.2, RRID : SCR_003199) along with log2FC and FDR values from each DE analysis. Genes were ranked according to the product of -log10(p value) multiplied by log2(FoldChange) in descending order.

### Weighted gene co-expression network analysis

Weighted gene co-expression network analysis was performed using the R package WGCNA (33) (v:1.71, RRID : SCR_003302) to identify groups (modules) of co-expressed genes using the gene expression data of the 74 SLE patients. Identified modules were correlated with the patients’ clinical SLEDAI-2K, and significant modules (p<0.05) were tested for functional enrichment using g:Profiler (RRID : SCR_006809) (34). Genes with a central role (Hub genes) in each of the significant modules were determined using the connectivity measure of Module Membership (MM > 0.8).

### LncRNA annotation and cis-gene identification

The LNCipedia (35) database (v:5.2) was used to identify lncRNAs in the DE gene list and hub gene list. cis-Genes (-10 kb upstream of gene start position, +10 kb downstream of gene end position) of the hub lncRNAs were extracted from the Ensembl (36) database (version 105, RRID : SCR_002344) using the R package biomaRt (37) (v:2.52.0, RRID : SCR_019214). cis-Elements were visualized using the packages Gviz (38) and karyoploteR (RRID : SCR_021824) (39).

### Gene-level correlation and RT-qPCR validation

The correlation of clinical SLEDAI-2K with the expression of the identified hub lncRNAs and of lncRNAs belonging to both ‘quiescent disease’ and ‘flare’ signature was tested in R using the Spearman coefficient and the function ‘cor.test’ with a significance threshold of p < 0.05. Healthy samples were assigned a SLEDAI-2K score of -1. The expression of hub lncRNAs significantly associated with SLEDAI-2K (p < 0.05) was validated in an independent cohort of 15 patients and 9 HC (Validation Cohort) using quantitative reverse transcription-polymerase chain reaction (RT-qPCR). RNA was isolated as mentioned above, and cDNA was created using PrimeScript RT-PCR Kit (#RR037A, Takara). Patient samples of the validation cohort were equally distributed in each activity group: five InaD, five IDA, and five HDA patients. GAPDH gene expression was used as baseline reference for calculating the relative expression of target genes. Experiments were performed on an Applied Biosystems QuantStudio 5 Real-Time PCR System using the KAPA SYBR Fast Universal Kit (#KK4602, Kapa Biosystems). Primer sequences are available in **Supplementary Table 3**. Spearman coefficient and function ‘cor.test’ with a significance threshold of p < 0.05 were used to evaluate clinical SLEDAI-2K correlation with ΔCt values of each gene in the validation cohort.

### Competing endogenous RNA network construction

LncACTdb 3.0 (40) was utilized to discover kidney-related ceRNA effects of lncRNAs validated by qRT-PCR. A list of mRNA and miRNA targets was retrieved for lncRNAs present in the database. To narrow down the list of potential mRNA targets of each lncRNA, only mRNAs which were part of the same module as their corresponding lncRNA were kept. To expand the list of possible ceRNA events, the miRNA lists (**Supplementary Table 4**) were used as input to miRWalk (RRID : SCR_016509) (41). Putative mRNA targets for all regions (3′UTR, 5′UTR, and CDS) were queried, and results were filtered for experimentally supported interactions as indicated by miTarBase (42) (**Supplementary Table 5**). Finally, the new list of potential mRNA targets was filtered, keeping only those present in the *Lightgreen* and *Lightyellow* modules and only triplets containing downregulated mRNAs (logFC <0 for LN vs. healthy differential expression analysis) (**Supplementary Table 6**). The network was visualized using packages igraph (RRID : SCR_019225) (43), qgraph (44), and ggraph (RRID : SCR_021239) (45) in R.

## Results

### Blood transcriptome profile of LN during quiescence and flare

Initially, we explored the transcriptome (both coding and non-coding) of LN patients and healthy controls (HC) using transcriptomic data from our discovery cohort and identified 5,899 DE genes between LN and HC (total LN signature), 39 DE genes between HDA vs. InaD patients (flare signature), and 4,490 DE genes between InaD vs. HC (quiescent disease signature) (**Figure 1B**). Using GSEA, we identified enriched biological processes and pathways, well established in SLE, such as interferon (IFN) type I response and interleukin (IL) production (i.e., IL1, IL2, IL8, and IL12) (46, 47). It was noted that response to type I IFN signaling is deregulated in quiescent disease and further contributes to flares. However, IL production seems to be involved only in quiescent disease and not in flares. It was also shown that complement activation, B-cell activation, and immunoglobulin (Ig) production processes were deregulated in flares only (**Figure 1C**). A higher number of genes and pathways (4,070 genes, 406 GO : BP terms) were found to be common between the “total LN” signature and the “quiescent disease” signature compared with genes and pathways shared between “total LN” and “flare” signature (13 genes, 22 GO : BP terms, **Figures 1D, E**). Overall, these results indicate that most molecules and signaling cascades are deregulated at quiescent disease, and only few pathways, such as IFN, complement, B-cell activation, and Ig production contribute to flares.

### LncRNA expression during quiescence and flare: biomarkers for flares

**Figure 2:**
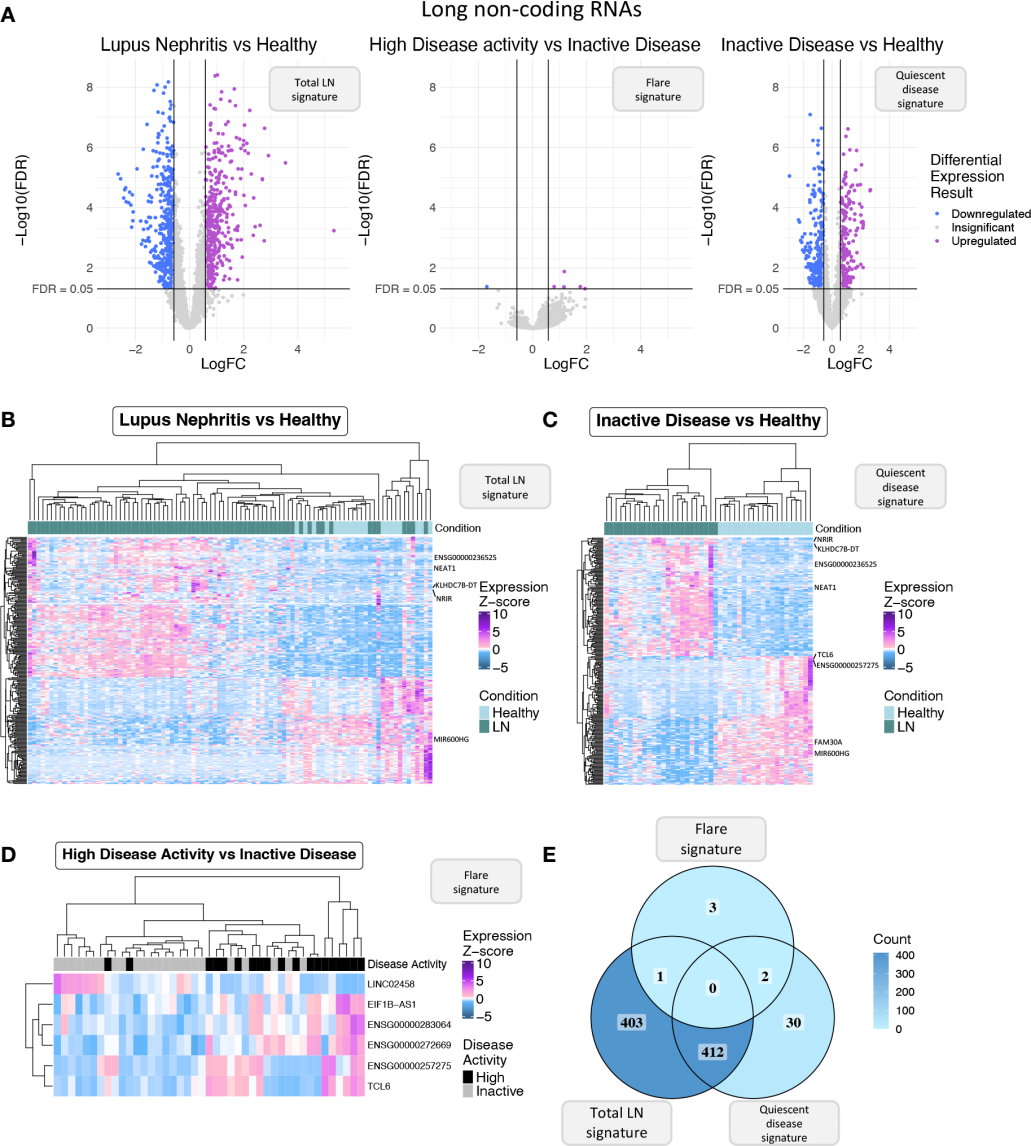
**(A)** Volcano plot of the long non-coding RNAs (lncRNAs) in each of the three differential expression analyses (DEA) performed comparing (left to right) LN patients vs. HC, HDA vs. InaD patients, and InaD patients vs. HC. Upregulated lncRNAs are colored violet, and downregulated lncRNAs are colored blue. LncRNAs not reaching our significance thresholds (|log2FC| >0.58 and FDR <0.05) are shown in gray. **(B)** Heatmap showing the expression profile of the top 250 lncRNAs with the highest absolute log 2 fold change value found as DE between LN and HC. Expression values were z-score normalized. Top annotation row shows the condition of each sample, colored green for LN patients and light blue for HC. **(C)** Heatmap showing the expression profile of the top 250 lncRNAs with the highest absolute log 2 fold change value found as DE between InaD patients and HC. Expression values were z-score normalized. Color scale of top annotation is the same as **(B)**. **(D)** Heatmap showing the expression profile of the six lncRNAs found as DE between HDA and InaD patients. Expression values were z-score normalized. Top annotation row shows the disease activity group of each sample with black representing HDA patients and gray representing InaD patients. **(E)** Venn diagram comparing the DE lncRNAs of each DEA. Color gradient corresponds to the term count in each compartment.

Next, we focused specifically on lncRNAs to explore how this transcript population is implicated in disease activity. We pinpointed 816 DE lncRNAs in the “total LN” signature, 6 DE lncRNAs in the “flare” signature, and 444 DE lncRNAs in the “quiescent disease” signature (**Figure 2A**). The expression of DE lncRNAs for each signature is shown in **Figures 2B-D**, and lncRNAs that have previously been associated with SLE such as NEAT1 and ENSG00000236525 are highlighted. Accordingly with the total transcriptome comparison, we observed more genes to be common between the “total LN” and “quiescent disease” signatures compared with the “total LN” and “flare” signatures (412 DE lncRNAs vs. 1 DE lncRNA, ENSG00000283064) (**Figure 2E**). This large number of deregulated LncRNAs in the “quiescent disease” signature suggests that lncRNAs may play a major role at LN onset. In contrast, the “flare” signature involves only six lncRNAs, suggesting that only few lncRNAs are involved in the transition from quiescence to flare. Noticeably, lncRNAs TCL6 and ENSG00000257275 were part of both “quiescent disease” and “flare” signatures, indicating that these genes undergo expression alterations both at the establishment of the disease and during flares. These data suggest that TCL6 and ENSG00000257275 are potential biomarkers able to discern between individuals under flare, quiescent disease and healthy state.

### LncRNAs play a central role in disease activity-associated pathways

**Figure 3:**
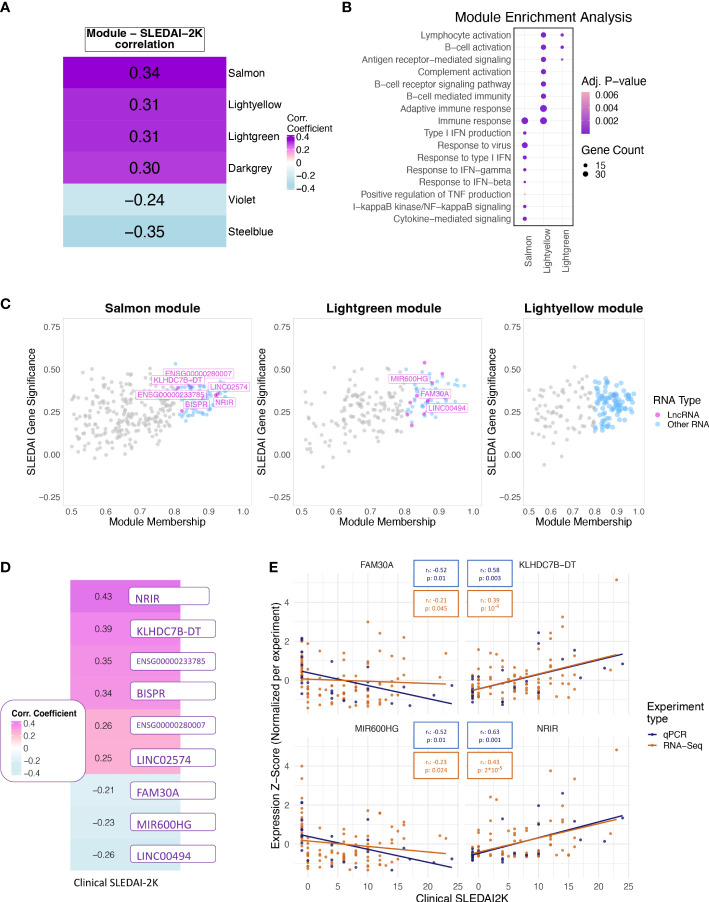
**(A)** Heatmap showing the correlation of the eigengene of each module found to be significantly correlated with SLEDAI-2K. Color corresponds to correlation level with purple for positive and blue for negative correlation. **(B)** Bubble plot of Gene Ontology terms found as significantly enriched in the top three correlated modules (*Lightgreen*, *Salmon*, *Lightyellow*). Color represents adjusted p-values, and size represents the number of genes related to a term found in each module. **(C)** Scatterplot of SLEDAI-2K Gene Significance against Module Membership for each gene in the (left to right) *Salmon*, *Lightgreen*, and *Lightyellow* modules. Genes with MM >0.8 (Hub genes) are shown in color, with violet for lncRNAs and blue for other RNA types. Genes with MM ≤0.8 (non-hub genes) are shown in gray. **(D)** Heatmap showing the correlation of the RNA-Seq-based expression values of the nine hub lncRNAs that were significant when tested using the Spearman correlation coefficient. Color corresponds to correlation level with purple for positive and blue for negative correlation. **(E)** Scatterplots of expression levels of FAM30A (top left), KLHDC7B-DT (top right), MIR600HG (lower left), and NRIR (lower right) normalized using z-score scaling per experiment type (qPCR, RNA-Seq) against SLEDAI-2K values. Boxes on top of each plot show the Spearman correlation coefficient and the associated p-value. Colors correspond to experiment type with blue for qPCR, gold for RNA-Seq.

To further explore the transcriptomic landscape related to disease activity, we performed a weighted gene co-expression network analysis (WGCNA) using patient transcriptomic data of the discovery cohort. WGCNA analysis identified 35 modules of co-expressed genes, six of which were significantly correlated with clinical SLEDAI-2K values (**Figure 3A**) and thus were labeled as disease-activity-related. This was followed by enrichment analysis to identify deregulated pathways in each module. In this analysis, the top 3 modules, *Salmon*, *Lightyellow*, and *Lightgreen*, were associated with IFN signaling, adaptive immune response, and B-cell signaling, respectively (**Figure 3B**). The last positively correlated module, *Darkgrey*, was enriched in DNA-protein binding, and the two negatively correlated modules, *Violet* and *Steelblue*, were enriched in transmembrane or electron transfer activity and calcium channel functions, respectively (**Supplementary Figure 1A**). To identify hub lncRNAs, we used the measure of module membership (MM), a measure that reflects the similarity in expression patterns between a gene and the rest of the genes in a module. A total of 18 hub lncRNAs were identified in *Salmon* (n=7), *Lightgreen* (n=10) and *Lightyellow* (n=1) modules (**Figure 3C**). *Darkgrey*, *Violet*, and *Steelblue* had no hub lncRNAs and were not further investigated (**Supplementary Figure 1B**). The lack of hub lncRNA in the last three modules suggests that lncRNAs are not involved in all disease activity-related processes, although their presence in the IFN- and adaptive-immunity-related modules highlights their pivotal position regulating key pathways involved in flares.

### Assessment of the biomarker potential of hub lncRNAs

To determine if each hub lncRNA could be useful as a potential biomarker of disease activity, we performed correlation analysis of lncRNA expression levels with SLEDAI-2K values using both patient samples and HC of the discovery cohort. The large number of DE genes and lncRNAs in the quiescent disease signature highlights the different state of patients at quiescence and HC. This difference should be reflected in our measure of disease activity in order to assess whether the biomarker can not only determine disease activity but also distinguish between HC and quiescent state patients. Therefore, we assigned HC a SLEDAI-2K value of -1 to differentiate them from the InaD patient group. We also included lncRNA ENSG00000257275 in the correlation analysis because it appeared along with hub lncRNA TCL6 in both of the “quiescent disease” and “flare” signatures, indicating a discriminatory potential that covers the complete spectrum of disease activity. Correlation at the gene level was significant for 9 out of 19 tested lncRNAs (**Figure 3D**). Six hub lncRNAs, NRIR, KLHDC7B-DT, ENSG00000233785, BISPR, ENSG00000280007, and LINC02574 (hub lncRNAs of the *Salmon* module), were positively correlated with disease activity, whereas FAM30A, MIR600HG, and LINC00494 (hub lncRNAs of the *Lightgreen* module) had a negative correlation. RT-qPCR experiments measuring gene expression in an independent validation cohort validated the positive correlation of NRIR and KLHDC7B-DT (**Figure 3E**), thus suggesting an inducing effect on flares. The negative correlation of MIR600HG and FAM30A was also validated (**Figure 3E**), implying that these lncRNAs may have some flare-inhibitory properties.

### Identification of cis-targets of significant lncRNAs

**Figure 4:**
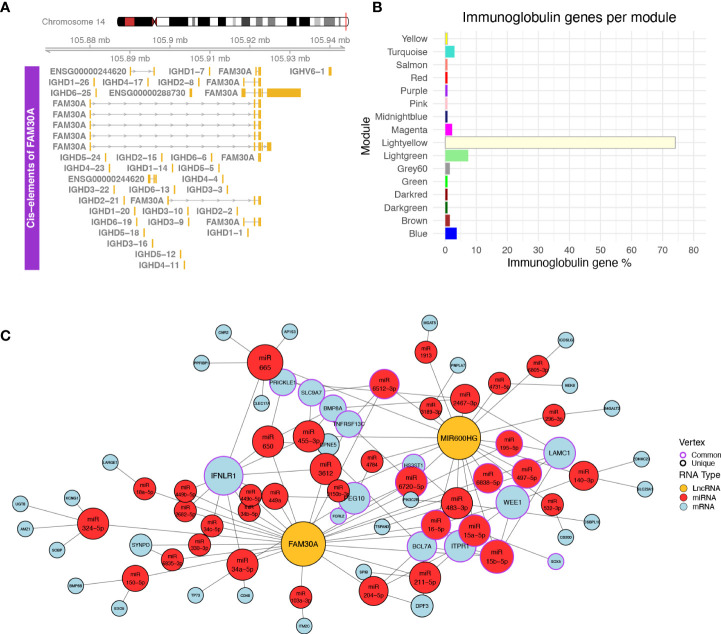
**(A)** Plot of the genomic region surrounding FAM30A. The genomic region depicted corresponds to 12 kbp upstream of the FAM30A start position and 12 kbp downstream its end position. Identified transcripts of genes found in the area are shown in gold (exons) connected by gray lines with arrows (introns). The exact position of the locus in the human genome is marked by the red line on the right side of the Chromosome 14 ideogram on the top of the figure. **(B)** Bar plot showing the percentage of immunoglobulin (IG) genes found in each WGCNA module. Each bar is colored according to the module name. **(C)** Network of ceRNA interactions of FAM30A and MIR600HG. Node fill color corresponds to RNA type with gold for LncRNA, red for miRNA, and light blue for mRNA. The node outline is colored depending on whether the node is connected to both FAM30A and MIR600HG (common—purple) or just one of the two lncRNAs (unique—black). Node size is a function of each degree with highly connected nodes shown as bigger points. The network layout was created using the *Davidson and Harels* simulated annealing algorithm of the igraph package.

Following the validation of NRIR, KLHDC7B-DT, MIR600HG, and FAM30A expression relative to disease activity, we investigated how these lncRNAs may exert their regulatory actions. We identified one cis-gene for NRIR, CMPK2, which was co-expressed with NRIR (belongs to the *Salmon* module) and three cis-genes for KLHDC7B-DT; SYCE3, ODF3B, and ENSG00000273272 (**Supplementary Figures 2A, B**). ODF3B and ENSG00000273272 belong to the *Salmon* module, whereas SYCE3 was not found in our dataset. We also identified two cis-genes in the vicinity of MIR600HG, STRBP, and MIR600 (**Supplementary Figure 2C**). Both MIR600HG and STRBP are members of the *Lightgreen* module. MIR600 is a miRNA and was not detected in our dataset. Finally, we identified 29 cis-genes ±10 kb of FAM30A, including the previously identified hub lncRNA ENSG00000244620 (MM = 0.835), a novel transcript ENSG00000288730, which is a hub RNA of the *Lightgreen* module (MM = 0.878), is not present in LNCipedia, and has no associated function, and 27 immunoglobulin heavy chain genes (IGH-) (**Figure 4A**). The presence of a high number of IGH-genes in the vicinity of FAM30A (**Figure 4A**), combined with the facts that a) the *Lightyellow* and *Lightgreen* modules contain 74% and 7% of immunoglobulin genes expressed in the dataset, respectively (**Figure 4B**), b) FAM30A has a high module membership in both *Lightgreen* (MM = 0.867) and *Lightyellow* (MM = 0.784) modules, and c) pathway enrichment of both modules is closely related to adaptive immunity, concludes that FAM30A is a gene with a significant role in both modules. Therefore, whereas most IGH-genes (81%) are assigned to the *Lightyellow* module, they have a strong probability of being cis-targets of FAM30A, rendering FAM30A a potential key regulator of immunoglobulin gene expression and antibody formation.

### Delineation of trans-effects of MIR600HG and FAM30A

LncRNAs can also have trans-regulatory effects, regulating the expression of distant genes. The competing endogenous RNA (ceRNA) hypothesis (48) describes such trans-regulation events where lncRNAs bind to miRNA and inhibit miRNA-guided degradation of mRNAs. We searched LncActDB, a database of experimentally validated ceRNA interactions, to identify ceRNA events NRIR, KLHDC7B-DT, MIR600HG, and FAM30A participate in. Data were available only for MIR600HG and FAM30A. Because LncActDB provides ceRNA interactions stratified by tissue, we focused on the 15 and 116 events identified respectively for MIR600HG and FAM30A in kidney tissue. Each event is defined by an affected mRNA and a list of miRNAs interacting with both the lncRNA and the mRNA. To verify the presence of these interactions, we used our WGCNA data and intersected the mRNA targets with the *Lightyellow* and *Lightgreen* modules. We identified only one common gene, B4GALT2, between LncActDB and the *Lightyellow* module. Thus, we further expanded the analysis *in silico* to identify putative ceRNA interactions based on our data. To achieve this, we used the lists of miRNAs that interact with MIR600HG (MIR600HG miRNAs, n = 53) and FAM30A (FAM30A miRNAs, n = 43) provided by LncActDB and used them as input to the miRNA target prediction tool miRWalk. After filtering using miTarBase information to include only experimentally supported interactions, miRWalk identified 3,071 interactions for MIR600HG miRNAs and 2,589 interactions for FAM30A miRNAs. We further refined the mRNA target list by removing mRNAs not included in the *Lightgreen* and *Lightyellow* modules. Finally, since MIR600HG and FAM30A are downregulated in LN patients, thus their miRNA-”sponging” effect would not be observed and miRNAs would be able to bind to their mRNA targets lowering their expression. To take this into account, we included only downregulated mRNAs of our “total LN” signature. Our final network consists of FAM30A, MIR600HG, 38 miRNAs, and 42 mRNAs of which 32 belong to the *Lightgreen* module and 10 belong to the *Lightyellow* module (**Figure 4C**). Network analysis revealed IFNLR1, an interferon-related mRNA, as the gene influenced the most by being targeted by 11 miRNAs. Interestingly, FAM30A and MIR600HG do not have a common way of regulating this mRNA. FAM30A can regulate IFNLR1 by interacting with nine different miRNAs (hsa-miR-34a-5p, hsa-miR-3612, hsa-miR-449a, hsa-miR-650, hsa-miR-2682-5p, hsa-miR-34b-5p, hsa-miR-34c-5p, hsa-miR-449b-5p, hsa-miR-449c-5p), whereas MIR600HG regulates IFNLR1 through two different miRNAs (hsa-miR-455-3p hsa-miR-665). This analysis reveals the impact of FAM30A and MIR600HG on multiple targets involved in adaptive immunity and provides a mechanism of regulation that features multiple parallel ways of targeting the same mRNA.

## Discussion

In this study, we used whole-blood RNA samples of LN patients and HC to explore the transcriptomic landscape of LN focusing on lncRNAs, the role of which is largely unexplored. DE and enrichment analyses confirmed the deregulation of pathways such as IFN response and IL-2 production, which have previously been reported to be associated with SLE (46, 47). Similar to previous studies, we define distinct LN-related signatures for quiescent disease and flares (49, 50). We focused on lncRNAs and the relationship of their gene expression levels with disease activity as measured using SLEDAI-2K and identified six DE lncRNAs in the “flare” signature, hinting that changes in lncRNA expression may be subtle during periods of exacerbated symptoms. This led us to use a different approach in order to determine lncRNAs with a key role in LN flares.

To this end, WGCNA was performed and six modules associated with disease activity were identified. Previous studies have reported an IFN-related module as the most significantly associated with disease status both in renal tissue and blood samples (51, 52). Consistent with these results, our findings show the direct positive correlation of the IFN-module with SLEDAI-2K. We also report that the adaptive immunity and B-cell receptor pathways appear to be significant contributors to high disease activity through the correlation of the relevant modules with SLEDAI-2K. There were 18 lncRNAs with a high module membership and a possible central role in regulating the disease-activity-related processes discovered, with nine of them showing a significant correlation of their expression levels with SLEDAI-2K. This correlation is an indication that hub lncRNAs could be utilized as blood-based biomarkers. Importantly, assigning a pseudo-SLEDAI-2K value of -1 to HC to distinguish them from InaD patients is a useful step to better evaluate their use as a biomarker, since a large number of DE lncRNAs were found in the quiescent disease signature supporting the previously reported observation that patients with achieved clinical remission may not always have achieved histological remission (53). Thus, with our approach, we also evaluate their potential use as biomarker to detect LN molecular activity present in clinical remission cases. Quantitative PCR experiments validated the significant association observed in the transcriptomic data for four of these lncRNAs, namely, NRIR (positive), KLHDC7B-DT (positive), FAM30A (negative), and MIR600HG (negative).

NRIR has been extensively investigated in previous studies in autoimmunity. In systemic sclerosis (SSc) (54), it was associated with the IFN score of SSc patients; in primary Sjogren’s syndrome (pSS), it was found to correlate with pSS disease activity levels (55); and in SLE, it was observed with substantially higher expression in SLE patients compared with HC (56). In the last study, researchers also correlated the expression levels of two IFN-stimulated genes, RSAD2 and USP18, with the SLEDAI-2K score of LN samples of their SLE cohort, although the correlation could not reach statistical significance for NRIR. We corroborate and expand their findings showing the significant relationship of NRIR expression levels with SLEDAI-2K. The second lncRNA, KLHDC7B-DT, has been studied previously in pancreatic ductal adenocarcinoma (57), where it was found to bind to the IL6 promoter region, and in psoriasis (58), where researchers showed a relationship between the lncRNA and ILF2, a T-cell-associated enhancer of the IL2 gene, with both genes being upregulated in lesional skin. Our novel finding suggests a central role of KLHDC7B-DT in LN disease activity. The third lncRNA, FAM30A, has previously been detected to interact with hub genes in a WGCNA analysis of a rheumatoid arthritis study (59), with our study revealing a similar central role of FAM30A in LN for the first time. Lastly, MIR600HG has been shown in a recent study to be differentially expressed between SLE patients and HC (60). The same study showed its co-expression with CD40LG, a gene encoding the CD40 ligand which is expressed on the surface of T cells and interacts with the CD40 antigen on the surface of B cells to signal B-cell activation. In light of another study (61) which has shown a direct positive correlation of CD40 expression and SLEDAI index in pediatric SLE patients, MIR600HG appears as a valid candidate for a blood-based biomarker of disease activity.

We also investigated the cis-effects of NRIR, KLHDC7B-DT, MIR600HG, and FAM30A, to identify a potential mechanism of action through which they influence disease activity. cis-Targets of NRIR and KLHDC7B-DT were defined as genes 10 kb upstream or downstream of the gene belonging to the IFN-module (*Salmon*). We detected CMPK2, a gene participating in IFN-dependent and IFN-independent antiviral immunity (62), as a potential cis-target of NRIR. CMPK2 has been reported as part of the SLE “flare” signature in a previous study (63), further linking NRIR’s locus to disease activity. For the KLHDC7B-DT gene, we identified three potential cis-targets, ENSG00000273272, ODF3B, and KLHDC7B. ENSG00000273272 has not been studied yet and no function is associated with this gene, ODF3B has been associated with SSc (64) and multiple sclerosis (65), and KLHDC7B has been linked to the IFN signaling pathway (66). cis-Targets of MIR600HG had to satisfy the 10-kb distance threshold and belong to the Lightgreen module. We identified STRBP, which a previous study using machine learning on SLE patient samples has shown to correlate with the expression levels of CD22, a surface marker of mature B cells (67, 68). The membership of MIR600HG and STRBP in a B-cell-related module and the previous association of STRBP with CD22 indicate the possibility of a B-cell-specific cis-regulation mechanism. The last validated lncRNA, FAM30A, is a hub gene of the *Lightgreen* module. We used the same distance threshold when determining cis-targets of FAM30A; however, due to the high module membership of FAM30A in both adaptive immunity (*Lightyellow*) and B-cell receptor (*Lightgreen*) modules, we considered genes as cis-targets if they belonged to either module. Excluding two hub RNAs (ENSG00000244620, ENSG00000288730) with no known function, cis-targets of FAM30A were 27 immunoglobulin heavy (IGH-) chain genes, members of the *Lightyellow* module. The high MM of FAM30A in the *Lightyellow* module suggests that FAM30A expression levels fluctuate with the same pattern as the IGH-genes’ expression levels, agreeing with a previous study (69) which uncovered a positive relationship of antibody titers with FAM30A expression levels after immune response to vaccination. Moreover, a recent tool investigating human gene co-expression using transcriptomic data from the GTEx consortium clearly illustrates the fact that FAM30A is highly co-expressed with immunoglobulin genes (70, 71). This evidence suggests a role of FAM30A in the regulation of IGH-genes and possibly antibody production.

Furthermore, we explored the trans-effects of FAM30A and MIR600HG through the *in silico* resources of LncActDB and miRwalk. Using the experimentally validated FAM30A-miRNA and MIR600HG-miRNA interactions of LncActDB and the experimentally validated miRNA–mRNA interactions of miRwalk, we created a ceRNA network of both lncRNAs. Intriguingly, their most prominent trans-target is interferon lambda (IFNλ) receptor 1 (IFNLR1), whose deficiency lowers the activation of immune cells and reduces organ damage in kidneys without affecting production of antibodies in murine lupus (72). Furthermore, another study marks IFNλ as an overlooked factor driving aberrancies in B cells in SLE and associates IFNLR1 with the expansion of the CD11c^+^CD21^-^ B-cell subset (73). Taking into account data from the ABIS Gene Viewer tool (74), which shows a B-cell- and plasmablast-specific expression of FAM30A (**Supplementary Figure 3**), the possibility of FAM30A exerting ceRNA effects on IFNLR1 and, thus, affecting B cells in LN becomes even more probable. This is an important finding considering the relationship of B-cell activity and antibody production with disease activity in LN. Given the negative correlation of FAM30A expression with SLEDAI-2K levels, it would be interesting to study the effect of FAM30A overexpression on disease activity, as it may reveal inhibitory effects of FAM30A on Ig gene transcription, resulting in lower antibody production and averting B-cell-activity-induced flares. Further research is needed to elucidate the exact mechanisms of FAM30A involvement in disease activity and assess the therapeutic potential of its overexpression.

This study elucidates the landscape of non-coding transcriptome in autoimmunity, by using total RNA sequencing to investigate the association of lncRNAs with disease activity in LN. Subsequent *in silico* analysis focusing on significant lncRNAs suggests the potential way of action of these lncRNAs. A potential limitation of our data is the use of SLEDAI-2K as a measure of disease activity as this index is not specific to renal activity of SLE. A validated index for lupus nephritis is not available. Additionally, the treatments the recruited patients received are another potential limitation, yet no pattern of medication effect was observed during exploratory analysis of our transcriptomic data, and sequencing results were experimentally replicated in an independent cohort.

In summary, using transcriptomic data from blood samples of LN patients and a network-based approach, we emphasize the key role of IFN pathway and reveal the importance of B-cell activity and antibody production in LN flares. Furthermore, our findings identified four lncRNAs, NRIR, KLHDC7B-DT (IFN-related), and MIR600HG, FAM30A (B-cell-related), as potential biomarkers of disease activity and central components of IFN signaling and adaptive immunity with a possible cis- and trans-effect on genes of the same pathways. Finally, we provide a network of trans-targets of FAM30A and MIR600HG, emphasizing the most prominent one, IFNLR1, and further corroborating the regulatory involvement of FAM30A in B-cell signaling, immunoglobulin gene expression, antibody production, and, consequently, in LN disease activity.

## Data availability statement

The datasets presented in this study can be found in online repositories. The names of the repository/repositories and accession number(s) can be found below: https://ega-archive.org/, EGAS00001007117.

## Ethics statement

The studies involving human participants were reviewed and approved by Attikon University Hospital Research Ethic Committee, Athens, Greece. Written informed consent to participate in this study was provided by the participants’ legal guardian/next of kin.

## Author contributions

GS and AF designed the experiments and analysis. GS performed the experiments, analyzed and interpreted data, generated figures, and wrote the manuscript. CL analyzed and interpreted data. NM participated in the library construction. DN, SF, and AB performed clinical evaluation of patients and provided human specimens. TM participated in the design of validation experiments and the interpretation of data. MG participated in sample preparation. DN, TM, MG, DB, and AF also participated in the editing of the manuscript. DB and AF acquired the grants and supervised the study, analysis of data, and the writing of the manuscript. All authors contributed to the article and approved the submitted version.

## Supplementary material

Supplementary Figure 1 **(A)** Bubble plot of Gene Ontology terms found as significantly enriched in the *Darkgrey*, *Violet* and *Steelblue* modules. Color represents adjusted p-values and size represents the number of genes related to a term found in each module. **(B)** Scatterplot of SLEDAI-2K Gene Significance against Module Membership for each gene in the (Left to right) *Darkgrey*, *Violet* and *Steelblue* module. Genes with MM>0.8 (Hub genes) are shown in color, with and blue for Other (Non-LncRNA) RNA types. Genes with MM ≤ 0.8 (Non-hub genes) are shown in grey.

Supplementary Figure 2 Plot of the genomic region surrounding NRIR **(A)**, KLHDC7B-DT **(B)** and MIR600HG **(C)**. The genomic region depicted corresponds to 12Kbp upstream of gene start position and 12Kbp downstream its end position. Identified transcripts of genes found in the area are shown in gold (exons) connected by grey lines with arrows (introns). The exact position of the locus in the human genome is marked by the red line on the Chromosome ideogram on the top of the figure.

Supplementary Figure 3 Cell-type-specific expression of FAM30A as indicated by ABIS (ABsolute Immune Signal) tool.
