# Supplementary figures and images for: A network-based approach reveals long non-coding RNAs associated with disease activity in lupus nephritis: key pathways for flare and potential biomarkers to be used as liquid biopsies
Source: Front Immunol. 2023 Jul 5;14:1203848. doi: 10.3389/fimmu.2023.1203848 (PMC10355154; doi:10.3389/fimmu.2023.1203848)

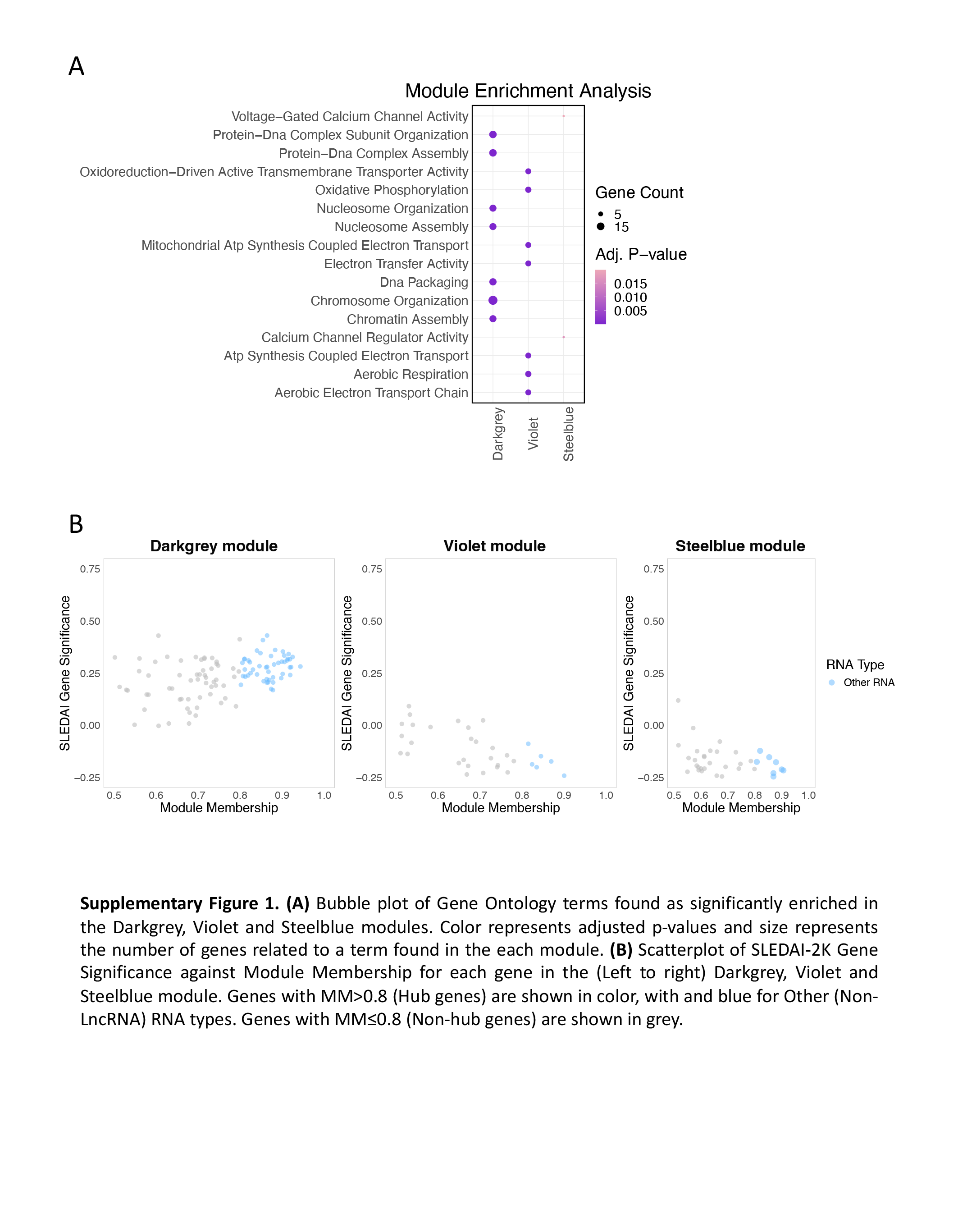

Supplement: Supplementary Figure 1 — (A) Bubble plot of Gene Ontology terms found as significantly enriched in the Darkgrey, Violet and Steelblue modules. Color represents adjusted p-values and size represents the number of genes related to a term found in each module. (B) Scatterplot of SLEDAI-2K Gene Significance against Module Membership for each gene in the (Left to right) Darkgrey, Violet and Steelblue module. Genes with MM>0.8 (Hub genes) are shown in color, with and blue for Other (Non-LncRNA) RNA types. Genes with MM ≤ 0.8 (Non-hub genes) are shown in grey. [file Image_1.tif]

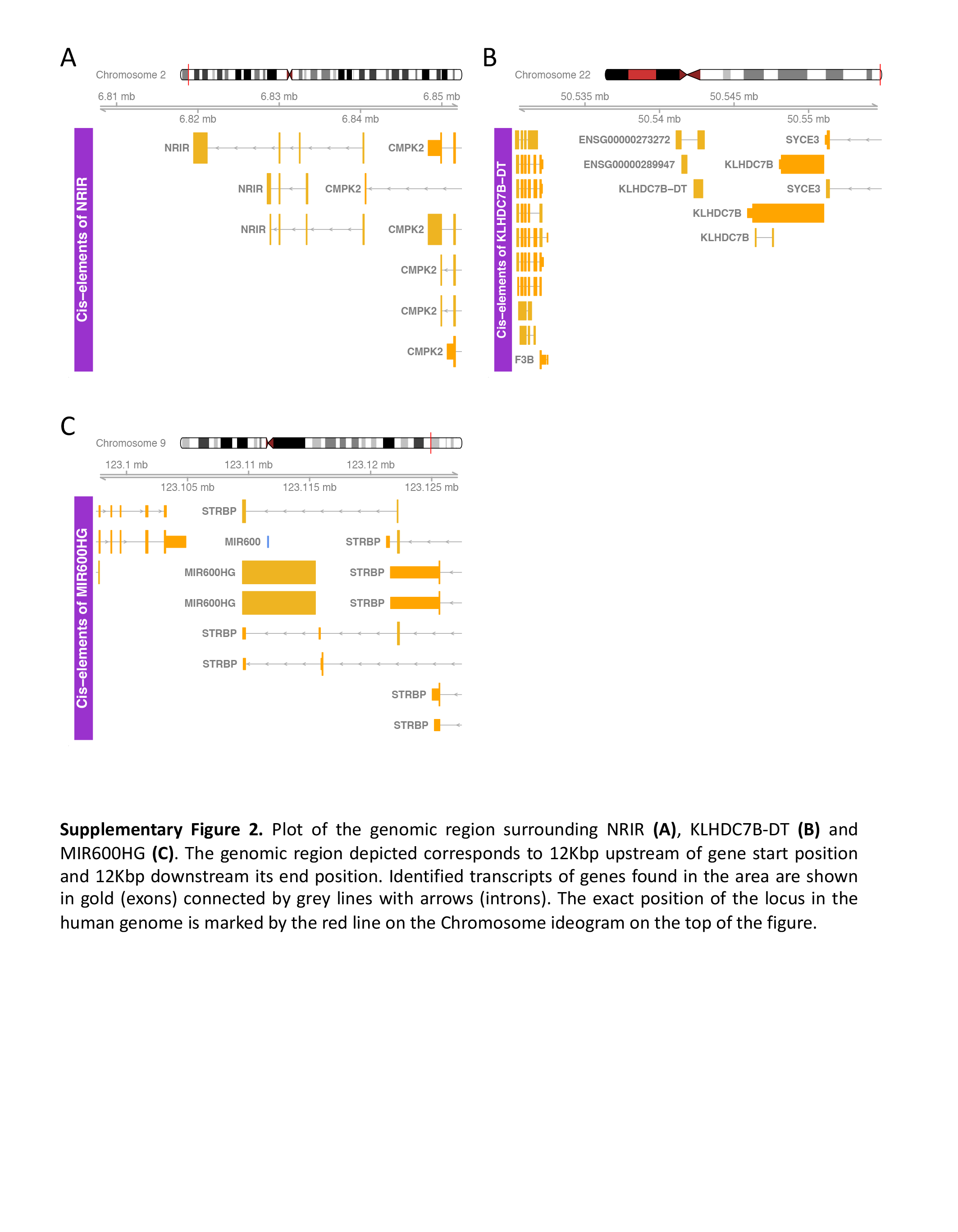

Supplement: Supplementary Figure 2 — Plot of the genomic region surrounding NRIR (A), KLHDC7B-DT (B) and MIR600HG (C). The genomic region depicted corresponds to 12Kbp upstream of gene start position and 12Kbp downstream its end position. Identified transcripts of genes found in the area are shown in gold (exons) connected by grey lines with arrows (introns). The exact position of the locus in the human genome is marked by the red line on the Chromosome ideogram on the top of the figure. [file Image_2.tif]

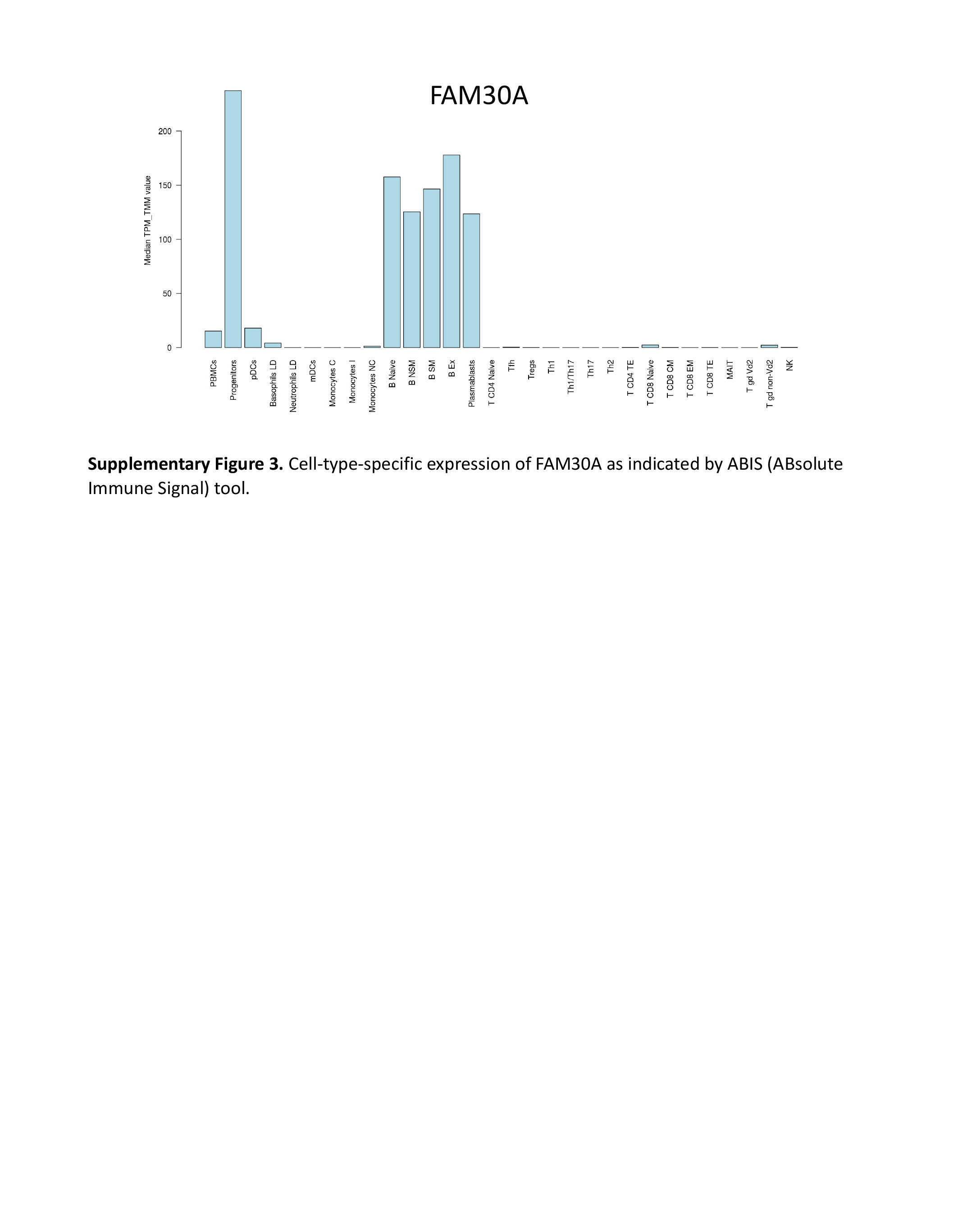

Supplement: Supplementary Figure 3 — Cell-type-specific expression of FAM30A as indicated by ABIS (ABsolute Immune Signal) tool. [file Image_3.tif]
