# Supplementary material for: A network-based approach reveals long non-coding RNAs associated with disease activity in lupus nephritis: key pathways for flare and potential biomarkers to be used as liquid biopsies
Source: Front Immunol. 2023 Jul 5;14:1203848. doi: 10.3389/fimmu.2023.1203848 (PMC10355154; doi:10.3389/fimmu.2023.1203848)
Supplement: Supplementary file 4 [file Table_1.docx]

**Supplementary Table 1.** Demographic and clinical characteristics of SLE patients and healthy individuals included in the discovery cohort of the study

|  | **SLE patients**  **(n = 74)** | **Healthy individuals**  **(n = 20)** |
| --- | --- | --- |
| **Gender** |  |  |
| Female | 65 (87.8%) | 19 (93.1%) |
| Male | 9 (12.2%) | 1 (6.9%) |
| **Race** |  |  |
| Caucasian | 87 (97.6%) | 20 (100%) |
| Other | 2 (2.4%) | 0 (0%) |
| **Age** (years) |  |  |
| Mean ± SD | 43.4 ± 14.1 | 41.9 ± 14.1 |
| Minimum-maximum | 9 – 82 | 20 – 68 |
| **ACR 1997 classification criteria** |  |  |
| Malar rash | 34 (45.9%) |  |
| Discoid rash | 9 (12.2%) |  |
| Photosensitivity | 27 (36.5%) |  |
| Mucosal ulcers | 17 (23%) |  |
| Arthritis | 62 (83.8%) |  |
| Serositis | 17 (23%) |  |
| Renal disease | 74 (100%) |  |
| CNS disease | 7 (9.5%) |  |
| Hematological | 34 (20.6%) |  |
| Immunological | 67 (90.6%) |  |
| Antinuclear antibodies (ANA) | 73 (98.7%) |  |
| *No. criteria* | 6.4 ± 1.2 |  |
| **Other disease characteristics** |  |  |
| Anti-DNA antibodies | 61 (82.4%) |  |
| Anti-phospholipid antibodies | 22 (29.7%) |  |
| Antiphospholipid syndrome | 11 (14.9%) |  |
| Neuropsychiatric SLE | 14 (18.9%) |  |
| Biopsy-proven nephritis | 65 (87.8%) |  |
| **SLE treatment** (at the time of blood sampling) | |  |
| Glucocorticoids | 51 (68.9%) |  |
| Hydroxychloroquine | 55 (74.3%) |  |
| DMARDs (methotrexate, azathioprine) | 20 (27%) |  |
| Mycophenolate mofetil | 18 (24.3%) |  |
| Cyclophosphamide | 12 (16.2%) |  |
| Biological treatment (rituximab/belimumab) | 10 (13.5%) |  |
| **SLE activity** (at the time of blood sampling) |  |  |
| ***Clinical SLEDAI-2K*** ^1^ | 7.6 ± 5.8 |  |
| cSLEDAI-2K = 0 (remission) | 12 (16.2%) |  |
| cSLEDAI-2K = 1–4 (mild activity) | 12 (16.2%) |  |
| cSLEDAI-2K = 5–11 (moderate activity) | 31 (41.9%) |  |
| cSLEDAI-2K ≥12 (severe activity) | 19 (25.7%) |  |
| ***Actively involved organs/domains*** ^2^ |  |  |
| General/constitutional | 20 (27%) |  |
| Mucocutaneous | 25 (33.8%) |  |
| Neurological | 4 (5.4%) |  |
| Musculoskeletal | 43 (58.1%) |  |
| Cardiorespiratory | 2 (2.7%) |  |
| Vasculitis (skin/GI) | 0 (0%) |  |
| Renal | 34 (49.4%) |  |
| Hematology | 3 (4%) |  |
| ***Immunological activity*** ^3^ |  |  |
| All patients | 37 (50%) |  |
| Within patients with remission (n=28) | 5 (17.8%) |  |
| Within patients with active SLE (n=46) | 32 (69.6%) |  |

^1^ SLEDAI-2K is based on the presence of 24 descriptors in nine organ systems over the preceding 30 days. Descriptors of SLEDAI-2K are documented as present or absent. Each of the descriptors has a weighted score and the total score of SLEDAI-2K is the sum of all 24 descriptor scores. The total SLEDAI-2K score falls between 0 and 105, with higher scores representing higher disease activity. Clinical SLEDAI-2K (cSLEDAI-2K) excludes the immunological activity descriptors which contribute a score of 4.

^2^ According to the BILAG classification (*Ann Rheum Dis*. 1996; 55:756–60)

^3^ Defined as low serum C3/C4 and/or increased anti-dsDNA concentrations
