# Supplementary material for: A network-based approach reveals long non-coding RNAs associated with disease activity in lupus nephritis: key pathways for flare and potential biomarkers to be used as liquid biopsies
Source: Front Immunol. 2023 Jul 5;14:1203848. doi: 10.3389/fimmu.2023.1203848 (PMC10355154; doi:10.3389/fimmu.2023.1203848)
Supplement: Supplementary file 5 [file Table_2.docx]

**Supplementary Table 2.** Demographic and clinical characteristics of SLE patients and healthy individuals included in the validation cohort of the study

|  | **SLE patients**  **(n = 15)** | **Healthy individuals**  **(n = 9)** |
| --- | --- | --- |
| **Gender** |  |  |
| Female | 13 (86.7%) | 8 (88.9%) |
| Male | 2 (13.3%) | 1 (11.1%) |
| **Race** |  |  |
| Caucasian | 15 (100%) | 10 (100%) |
| Other | 0 (0%) | 0 (0%) |
| **Age** (years) |  |  |
| Mean ± SD | 39.3 ± 12.5 | 42 ± 12.7 |
| Minimum-maximum | 20 – 70 | 25 – 62 |
| **ACR 1997 classification criteria** |  |  |
| Malar rash | 10 (66.7%) |  |
| Discoid rash | 0 (0%) |  |
| Photosensitivity | 12 (80%) |  |
| Mucosal ulcers | 3 (20%) |  |
| Arthritis | 15 (100%) |  |
| Serositis | 3 (20%) |  |
| Renal disease | 15 (100%) |  |
| CNS disease | 0 (0%) |  |
| Hematological | 7 (46.6%) |  |
| Immunological | 12 (80%) |  |
| Antinuclear antibodies (ANA) | 15 (100%) |  |
| *No. criteria* | 6.1 ± 1.4 |  |
| **Other disease characteristics** |  |  |
| Anti-DNA antibodies | 11 (73.3%) |  |
| Anti-phospholipid antibodies | 2 (13.3%) |  |
| Antiphospholipid syndrome | 0 (0%) |  |
| Neuropsychiatric SLE | 0 (0%) |  |
| Biopsy-proven nephritis | 14 (93.3%) |  |
| **SLE treatment** (at the time of blood sampling) | |  |
| Glucocorticoids | 11 (73.3%) |  |
| Hydroxychloroquine | 11 (73.3%) |  |
| DMARDs (methotrexate, azathioprine) | 4 (26.6%) |  |
| Mycophenolate mofetil | 6 (40%) |  |
| Cyclophosphamide | 2 (13.3%) |  |
| Biological treatment (rituximab/belimumab) | 0 (0%) |  |
| **SLE activity** (at the time of blood sampling) |  |  |
| ***Clinical SLEDAI-2K*** ^1^ | 10.1 ± 7 |  |
| cSLEDAI-2K = 0 (remission) | 1 (6.7%) |  |
| cSLEDAI-2K = 1–4 (mild activity) | 4 (18%) |  |
| cSLEDAI-2K = 5–11 (moderate activity) | 5 (40.4%) |  |
| cSLEDAI-2K ≥12 (severe activity) | 5 (27%) |  |
| ***Actively involved organs/domains*** ^2^ |  |  |
| General/constitutional | 3 (20%) |  |
| Mucocutaneous | 6 (40%) |  |
| Neurological | 0 (4.5%) |  |
| Musculoskeletal | 12 (80%) |  |
| Cardiorespiratory | 1 (6.7%) |  |
| Vasculitis (skin/GI) | 0 (0%) |  |
| Renal | 8 (53.3%) |  |
| Hematology | 1 (6.7%) |  |
| ***Immunological activity*** ^3^ |  |  |
| All patients | 7 (46.7%) |  |
| Within patients with remission (n=1) | 0 (0%) |  |
| Within patients with active SLE (n=14) | 7 (50%) |  |

^1^ SLEDAI-2K is based on the presence of 24 descriptors in nine organ systems over the preceding 30 days. Descriptors of SLEDAI-2K are documented as present or absent. Each of the descriptors has a weighted score and the total score of SLEDAI-2K is the sum of all 24 descriptor scores. The total SLEDAI-2K score falls between 0 and 105, with higher scores representing higher disease activity. Clinical SLEDAI-2K (cSLEDAI-2K) excludes the immunological activity descriptors which contribute a score of 4.

^2^ According to the BILAG classification (*Ann Rheum Dis*. 1996; 55:756–60)

^3^ Defined as low serum C3/C4 and/or increased anti-dsDNA concentrations
