# Supplementary material for: A network-based approach reveals long non-coding RNAs associated with disease activity in lupus nephritis: key pathways for flare and potential biomarkers to be used as liquid biopsies
Source: Front Immunol. 2023 Jul 5;14:1203848. doi: 10.3389/fimmu.2023.1203848 (PMC10355154; doi:10.3389/fimmu.2023.1203848)
Supplement: Supplementary file 6 [file Table_3.docx]

**Supplementary Table 3.** Primer sequences of long non-coding RNAs used in qPCR validation experiments.

| Gene | Forward | Reverse |
| --- | --- | --- |
| NRIR | TCCAGTGAAGACTTCCTCTGGA | TGCAGTGAGCCAATATCGCA |
| KLHDC7B-DT | TGTCATCTGCCCCGTGAAAT | TGGGAGAATGGGAACTGAGC |
| ENSG00000233785 | ACCAAAGGAATGTGAGCTCGT | GGGAAATCTCTCGCCAGGAG |
| BISPR | CCCAACACTTAGGCAGGAGG | ACAGAGACACAGATGCTGCC |
| ENSG00000280007 | AGTAGATGGGGAAGGGCTGG | GCTCCTAATGTCGCTCGTGA |
| LINC02574 | CTTCCCCAGGATCCCTTGG | GAGCCGAGATCGCACCATT |
| FAM30A | TGGGTCACAGGATATGCACG | CGAGGACCTTCTTGCTCCTC |
| MIR600HG | GGAGGTGGAAGCAACTACCC | TCAACGTGCCTAGAAACCCC |
| LINC00494 | ACGTCATACCTCAAGGCTGC | TCTGAGGAGTGATGAGGCCT |
